# *In vitro* reconstitution of substrate S-acylation by the zDHHC family of protein acyltransferases

**DOI:** 10.1098/rsob.210390

**Published:** 2022-04-13

**Authors:** R. Elliot Murphy, Anirban Banerjee

**Affiliations:** Section on Structural and Chemical Biology of Membrane Proteins, Neurosciences and Cellular and Structural Biology Division, Eunice Kennedy Shriver National Institute of Child Health and Human Development, National Institutes of Health, Bethesda, MD, USA

**Keywords:** S-acylation, *in vitro* reconstitution, zDHHC enzyme, membrane protein structure, membrane enzyme, protein lipidation

## Abstract

Protein S-acylation, more commonly known as protein palmitoylation, is a biological process defined by the covalent attachment of long chain fatty acids onto cysteine residues of a protein, effectively altering the local hydrophobicity and influencing its stability, localization and overall function. Observed ubiquitously in all eukaryotes, this post translational modification is mediated by the 23-member family of zDHHC protein acyltransferases in mammals. There are thousands of proteins that are S-acylated and multiple zDHHC enzymes can potentially act on a single substrate. Since its discovery, numerous methods have been developed for the identification of zDHHC substrates and the individual members of the family that catalyse their acylation. Despite these recent advances in assay development, there is a persistent gap in knowledge relating to zDHHC substrate specificity and recognition, that can only be thoroughly addressed through *in vitro* reconstitution. Herein, we will review the various methods currently available for reconstitution of protein S-acylation for the purposes of identifying enzyme–substrate pairs with a particular emphasis on the advantages and disadvantages of each approach.

## Graphical abstract

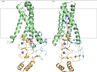

## 1. Introduction

Protein lipidation is the cellular process by which a lipid moiety is covalently attached, either co- or post-translationally, to a protein. The hydrophobic properties of the modification often confer profound changes to a protein's stability, subcellular localization and overall function. The lipid moiety can take the form of a fatty acyl molecule, isoprenoid or cholesterol and can be attached to any number of sites on the protein, including the amino terminus and the nucleophilic side chains of cysteine, lysine and serine residues [1]. The most prevalent form of protein lipidation found in nature is the attachment of a fatty acyl chain to a cysteine side chain via a reversible thioester bond, in a process known as S-acylation. Protein S-acylation has been demonstrated to play a key role in the regulation of multiple cellular processes, including membrane trafficking, protein secretion, signal transduction and apoptosis. This post translational modification, commonly referred to simply as palmitoylation, after the 16-carbon saturated fatty acid most often used in the reaction, was first discovered on the vesicular stomatitis virus glycoprotein nearly half a century ago [2].

For many years after the discovery of palmitoylated proteins, it remained unclear whether S-acylation occurs spontaneously or is enzymatically driven, as no enzyme had yet been identified. Erf2, the first protein to be identified with protein acyltransferase (PAT) activity, was discovered by genetic screening based on the activity of a palmitoylation dependent Ras protein [3]. The authors of this study identified a key region of Erf2, the cysteine rich domain containing a zDHHC motif (zDHHC-CRD), as being critical for the catalytic activity of the enzyme [4]. The zDHHC name refers to the highly conserved catalytic site, which is composed of the Asp-His-His-Cys sequence of amino acids. zDHHC-PATs have now been shown to be ubiquitous across all examined eukaryotes, with 7 eventually being discovered in yeast and 23 in mammals [5,6]. To date, only eukaryotes have been identified as having PATs. However, many bacterial and viral proteins require palmitoylation by host enzymes for proper function [7,8].

**Figure 1.**
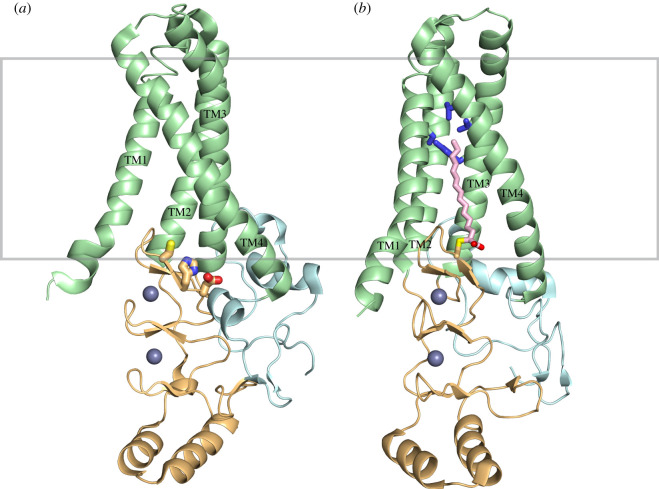
Crystal structures of human DHHC20 enzyme in cartoon representation. The four TMDs are shown in green, the cytoplasmic region containing the CRD is shown in orange, the C-terminal domain is shown in cyan and the Zn2 + ions are displayed as grey spheres. (*a*) DHHC20 apo structure with catalytic triad residues Asp^153^, His^154^ and Cys^156^ displayed as sticks from right to left. (*b*) DHHC20 2-BP bound structure with the ligand shown as pink sticks. Additionally, residues displayed as blue sticks represent those which close off the hydrophobic cavity that accommodates the acyl chain. These residues include Ser^29^, Tyr^181^, Val^185^ and Leu^213^.

The 23 human zDHHC-PATs represent a diverse family of enzymes with many common and unique features among them. Most members contain exactly four transmembrane domains (TMDs), with the CRD and catalytic site lying between the second and third helices on the cytosolic side of the membrane (depicted in figure 1). There are, however, many exceptions to this rule, such as zDHHCs 13, 17 and 23, which contain six TMDs with the zDHHC-CRD between the fourth and fifth helices. Additionally, zDHHCs 4 and 24 are predicted to have five TMDs and a catalytic site between helices 4 and 5 and 3 and 4, respectively. Bioinformatic analysis has revealed multiple other domains conserved across most zDHHC enzymes. These include the typically conserved, but enigmatic Asp-Pro-Gly (DPG) motif adjacent to TM2 on the cytoplasmic side of the membrane, as well as the Thr-Thr-xxx-Glu (TTxE) and the palmitoyltransferase conserved C-terminus (PaCCT) motif. The last two domains have documented functional relevance [9,10] and high-resolution structures of zDHHC15 and zDHHC20 indicate that the TTxE motif makes close contact with the catalytic site and that the PaCCT motif contributes to stability of the C-terminal domain [10]. The TMDs and zDHHC-CRD are highly conserved across the zDHHC family, but the N- and C-terminal domains tend to be highly variable in length and sequence. These variable regions are probably the primary driver of protein substrate specificity. A subset of zDHHC enzymes contain individual motifs with well-established propensity for protein–protein interactions, such as the ankyrin (ANK) repeats within the N-terminal domains of zDHHC13 and zDHHC17. A sizable subset, including zDHHC3, 5, 7, 8, 14, 16, 17, 20 and 21, have been shown to contain PDZ binding domains at their C-terminus with many of them playing a clear role in substrate recruitment [11–14].

## 2. zDHHC structure and catalytic mechanism

In 2018, our laboratory published the first structures of zDHHC enzymes, addressing major knowledge gaps in the field of protein palmitoylation. Specifically, the high-resolution crystal structures of zDHHC20 (figure 1*a*) and zDHHC15 (not shown) allowed for a deeper functional and mechanistic understanding of the protein family, as a whole. The structures revealed that the predicted four transmembrane helices form a tepee-like topology with very little lumenal exposure at the ‘top' of the tepee and a sizable cytosolic domain containing the zDHHC-CRD at the opposite end. The active site in zDHHC20, taking the form of a catalytic triad comprising the titular residues Asp153, His154 and Cys156, is located at the cytosolic interface (shown as sticks in figure 1*a*). The CRD, which forms a series of three stacked β-hairpins, coordinates two Zn^2+^ ions in a zinc finger-like arrangement. However, the active site Cys does not participate in Zn^2+^ coordination and neither of the ions appear to be directly involved in the catalytic mechanism. The Zn^2+^ ions are probably necessary for stabilizing the structure of the CRD as well as proper positioning of the catalytic Cys residue. These studies also revealed that, in the context of zDHHC20, the C-terminal domain packs tightly against both the zDHHC-CRD domain as well as with TM2, TM3 and TM4.

**Figure 2.**
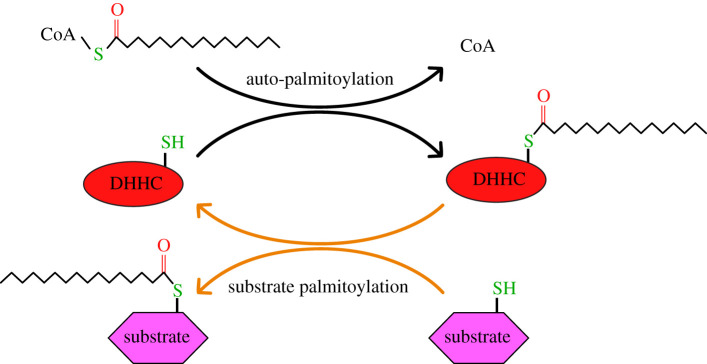
Putative mechanism of zDHHC substrate palmitoylation. The enzyme first undergoes autoacylation before transferring the fatty acid onto an exposed cysteine residue on the substrate protein in a two-step process.

Both the zDHHC20 and zDHHC15 structures revealed the presence of a hydrophobic cavity created by all four transmembrane helices, extending into the lipid bilayer from the cytoplasmic side of the membrane in what appears to be an ideal binding pocket for the acyl chain. This cavity and the positioning of the catalytic cysteine suggested a structural model for the proposed two-step enzymatic mechanism, depicted in figure 2, whereby the zDHHC enzyme is first autoacylated before transferring the fatty acid to the protein substrate. In order to establish whether the observed cavity could accommodate an acyl chain bound to the catalytic cysteine, our group crystallized zDHHC20 covalently bound to 2-bromopalmitate (2-BP), a known universal inhibitor of zDHHCs (structure displayed in figure 1*b*). 2-BP is an analogue of palmitic acid which contains a bromine bound to the α-carbon. The electronegativity of the bromine makes the α-carbon an ideal soft electrophile for the sulfhydryl group of the active site cysteine. Unlike the canonical reaction with palmitoyl-CoA, the complex formed by this reaction is irreversible [15]. This allowed for the clear visualization of the acyl chain within the binding pocket, providing insight into the mechanism of acyl-CoA binding and selectivity. It was observed that Trp^158^ and Phe^171^ are positioned at the base of the acyl chain near the head group, possibly as a means of positioning the thioester of palmitoyl-CoA for a nucleophilic attack by the adjacent Cys. These are two of the most highly conserved residues among the zDHHC family and their mutation to Ala results in severe abrogation of enzymatic activity. It was observed that the cavity is closed off at the opposite end by the presence of a hydrogen bond between Tyr^181^ and Ser^29^. These two residues, along with Val^185^ and Leu^213^, form the barrier at the tip of the hydrophobic cavity and are depicted as blue sticks in figure 1*b*. Mutation of the bulky Tyr^181^ residue to Ala resulted in a mutant of zDHHC20 with a preference for longer chain fatty acids compared to the wild-type enzyme, presumably due to the extra space available in the cavity allowing for the accommodation of larger acyl chains. Likewise, when Ser^29^ is mutated to a larger amino acid like Phe, the acyl-CoA selectivity shifts toward those with shorter fatty acid chains.

## 3. Diverse methodology of zDHHC substrate identification

**Figure 3.**
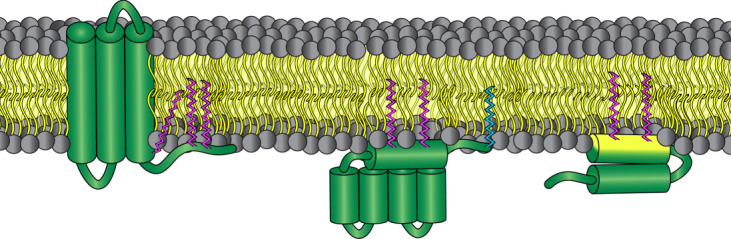
Categories of acylated proteins. Depiction of three types of acylated proteins categorized by the manner in which they associate with the membrane prior to acylation. From left to right: integral membrane protein (magenta zig-zags represent palmitoyl groups), proteins which associate with the membrane through preliminary PTMs (cyan zig-zag represents an N-terminal myristoyl group), and proteins which associate with the membrane through specific membrane-interaction domains (yellow protein surface represents the hydrophobic side of an amphipathic helix).

With 23 enzymes identified in eukaryotes, the zDHHC family is large and diverse. However, with over 10 000 unique proteins identified as possible zDHHC substrates [16], it is likely that each individual enzyme is capable of modifying hundreds of different substrate proteins. A clear conception of the mechanisms of substrate recruitment and specificity is an essential step toward understanding the critical role S-acylation plays across multiple aspects of cellular physiology and will bring us closer to the ultimate goal of targeting S-acylation for discovery of new therapies against human diseases. As mentioned above, there are a handful of zDHHC enzymes with domains shown to be involved in substrate recruitment, such as the ANK domains of zDHHC13 and zDHHC17. However, in the context of the zDHHC family as a whole, these cases represent an exception to the rule rather than the rule itself. In contrast to other PTMs, such as glycosylation or N-myristoylation, there is no consensus amino acid sequence or known structural motif which can be used to accurately predict bona fide S-acylation sites. Any cytosolically exposed cysteine residues of a transmembrane protein or membrane associated protein represents a potential site of S-acylation, but predicting which of the 23 zDHHC enzymes are capable of acting on those residues requires experimental dissection. As a consequence of their confinement within the membrane, zDHHC enzymes can only act upon proteins in close proximity to the membrane prior to palmitoylation. Consequently, there exists three known protein types which are susceptible to S-acylation by zDHHC enzymes: integral membrane proteins, such as the spike proteins from influenza and coronaviruses [8], as well as a host of mammalian proteins with human disease relevance including multiple G-protein-coupled receptors [17] and ion channels [18]; proteins which associate with the membrane via additional PTMs like the C-terminally farnesylated Ras proteins [19] or the N-terminally myristoylated flotillin-2 [20] and p59fyn [21]; and lastly, proteins such as GobX that associate with the membrane intrinsically through electrostatic or hydrophobic interactions [22]. A generalized example of each of these categories of palmitoylated protein is depicted in figure 3.

Recent years have seen the development of numerous methods for identifying zDHHC enzymes responsible for the S-acylation of a given substrate. These methods can be broken down into three constituent parts: the system in which the experiment is conducted, the experimental variable and the detection methods. There are essentially three ‘systems' in which the experiment can be conducted: *in vivo*, *in vitro* and *in cellulo*. *In cellulo* is used here to differentiate experiments which are conducted in cell culture versus *in vitro*, which are conducted essentially in ‘test tubes' with extracted or purified proteins. The ‘variable' aspect of the experiment refers to what is being changed in the system in order to differentiate the enzymes capable of palmitoylating a certain substrate versus those that are not. This can be done by knocking down/knocking out a gene coding for a particular zDHHC or, alternatively by co-over expression of the enzyme along with the substrate protein of interest. Lastly, there are a large number of techniques which have been developed for the detection of palmitoylated protein such as radiolabelling, click chemistry, and resin assisted capture (RAC) to name just a few. The different techniques used in each of these three aspects of the experiment carry their own advantages and disadvantages, examples of which will be discussed in detail in this section.

In terms of choosing a ‘system' for a given experiment, *in vivo* methods come with the obvious benefit of having all proteins of interest functioning normally within their native environments. However, a significant drawback to these experiments is that the details are often unresolved, meaning that they function more as jumping-off points for further investigation. One representative study set out to determine the role that zDHHC17 plays in synaptic signalling and plasticity through the generation of zDHHC17 knockout mice [23]. Therein, the authors observed major neurophysiological deficits associated with the zDHHC17 knockout. Single zDHHC knockout can provide valuable information about which organ systems are dependent on a particular enzyme and may even indicate particular protein–protein interaction networks or signalling pathways which the zDHHC may be involved in. The severity of any observed phenotypes or lack thereof could also shed light on the role of zDHHC substrate overlap in these pathways. Interestingly, it has been previously demonstrated that substrate-specific palmitoylation levels can be assessed and compared between knockout and wild-type mice [24]. In this study, palmitoylation assays were performed by immunoprecipitation of candidate proteins extracted from knockout and wild-type mouse brain tissue. Studies of this nature are rare due to their inherent complexity but provide valuable insight into role that palmitoylation plays in certain disease states and deserve further exploration.

While *in vivo* studies are useful for identifying the physiological effects caused by the loss of zDHHC function, they are not currently seen as a convenient or broadly applicable method for the identification of substrates for a particular zDHHC. The most common techniques for that purpose typically involve the much more simplified and controlled setting of an *in cellulo* or *in vitro* system. An *in cellulo* assay often involves the systematic knockdown or knockout of zDHHC enzymes individually, using techniques such as siRNA or CRISPR/Cas9 respectively, followed by detection of the levels of palmitoylation on a given substrate or some other phenotypic response. A decrease in detectable palmitoylation levels would indicate that the substrate's palmitoylation is dependent on the presence of the targeted zDHHC, which could arise out of a direct substrate-enzyme relationship but not necessarily so. One such study used an siRNA screen to determine which Golgi-resident zDHHCs were capable of palmitoylating tetraspanins CD9 and CD151 [25]. Only siRNA knockdown of zDHHC2 resulted in significant decrease in palmitoylation of the target proteins. A popular alternative to this method is to coexpress individual zDHHCs together with the target substrate and determining candidate zDHHCs by monitoring the ones that lead to an increase in the palmitoylation of the target. One of the first applications of this method was to identify the subset of zDHHC enzymes capable of palmitoylating PSD-95, a major regulator of synaptic activity [26]. Ideally, concomitant use of siRNA screens and overexpression screens can be used for cross validation and to pinpoint any false or misleading results. Lakkaraju *et al*. [27] identified zDHHC5 and zDHHC6 as effectors of calnexin palmitoylation in an siRNA knockdown screen. However, further analysis revealed that the zDHHC5 knockdown affected palmitoylation of endogenous but not ectopically expressed calnexin. After performing a follow-up overexpression screen and observing that only zDHHC6 affected calnexin palmitoylation, the group hypothesized that zDHHC6 may be palmitoylated by zDHHC5, and that this resulted in a false positive result in the knockdown screen.

## 4. Methods for detection of protein palmitoylation

**Figure 4.**
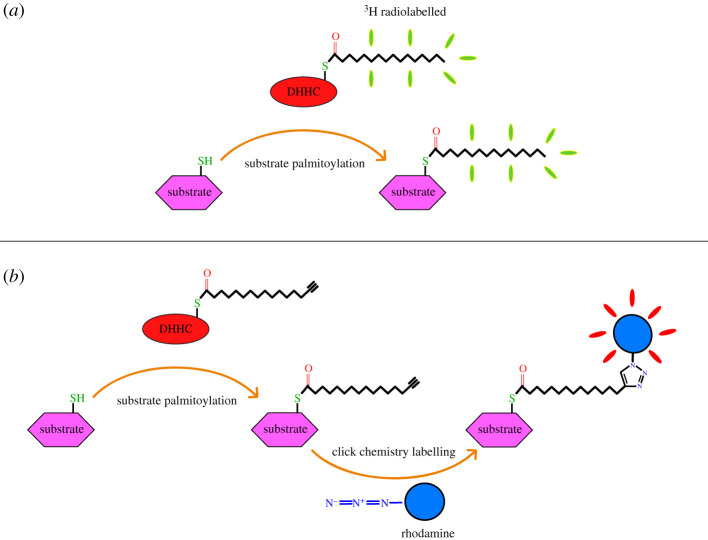
Methods for detecting substrate S-acylation. (*a*) radiolabelled acyl chains, once transferred for the substrate, can be detected by fluorography or scintillation spectroscopy. (*b*) Commercially available alkyne analogues of fatty acids can be detected after acyltransfer by performing a subsequent click chemistry reaction, followed by direct detection of the attached fluorophore.

Over the past several decades, many different methods have been used for the visualization and quantification of protein S-acylation. One of the earliest techniques used for this purpose, involves the incorporation of radiolabelled [^3^H]palmitic acid (depicted in figure 4*a*) [28]. In order to investigate the S-acylation of specific substrates, the experiment must either be performed *in vitro* using purified substrates [29] or, if purification is infeasible, the substrate can be immunoprecipitated from cell lysate after palmitoylation *in cellulo*. One advantage here is the ability to track rates of S-acylation and deacylation using pulse/chase metabolic labelling experiments [30]. However, due to the many complications that come with this method, such as exposure times of up to several weeks or the need to work with radioactive substrates [30,31], many in the field have pivoted to alternative methods using non-radioactive chemical probes [32].

One such alternative is the use of fatty acid analogues with small, reactive groups, such as an alkyne or an azide at the methyl end of the fatty acid chain. After the zDHHC reaction is carried out, either *in vitro* or *in cellulo*, a fluorescent moiety is attached to the fatty acid analogue by a bioorthgonal chemical technique such as a click chemistry reaction (depicted in figure 4*b*). The degree of substrate S-acylation can subsequently be visualized and quantified by in-gel detection or HPLC based analysis. The reactive fatty acid analogues can be obtained in the form of a fatty acid or acyl-CoA, depending on the application. Assays conducted *in cellulo* necessitate the use of fatty acid analogues which can be metabolically incorporated into the cell cultures and activated into fatty acyl-CoA [33], while *in vitro* assays require the acyl chain in the form of fatty acyl-CoA [34,35]. These detection methods are well suited for experiments in which the protein substrate is known or hypothesized.

Fluorescently labelled fatty acid analogues such as {N-[(7-nitro-2-1,3- benzoxadiazol-4-yl)-methyl]amino} palmitoyl-CoA (NBD-palmitoyl-CoA), are also commercially available and allow for the detection of palmitoylation without the need for secondary click chemistry reactions. This compound, easily incorporated into *in vitro* assays, can be used to monitor substrate palmitoylation by in gel fluorescence [10]. However, the bulky addition of NBD to the acyl chain excludes it from being used in any *in cellulo*-based assays and could conceivably reduce its accessibility to many zDHHC enzymes, particularly those which discriminate against fatty acids of greater chain length.

Another set of methods that do not require the use of fatty acyl-anologues, are the so-called ‘hydroxylamine switch' methods, which are generally well suited for either *in vivo* or *in cellulo* assays [36]. The two most commonly used approaches in this category are acyl-RAC and acyl biotin exchange (ABE). Both detection methods involve the initial reduction of disulfides with a reducing agent such as TCEP followed by the blocking of exposed thiol groups with a sulfhydryl reactive compound like a maleimide or methyl methanethiosulfonate [37]. Samples are then treated with hydroxylamine, which aggressively hydrolyses all thioesters; i.e. all S-acylated cys residues. Exposed thioesters are then either conjugated with pyridyl-disulfide biotin for enrichment by streptavidin-resin for ABE [37] or they may be captured directly by pyridyl-disulfide resin in the acyl-RAC method [38]. These techniques lend themselves well to a global approach for identifying unknown substrates of a particular zDHHC. However, they can also work well in a targeted approach if the protein/s of interest contain tags for purification or if they are able to be detected directly by western blot [39].

## 5. *In vitro* reconstitution of protein substrate S-acylation

Protein S-acylation is orchestrated by a complex network of acylating and de-acylating enzymes, all with varying degrees of substrate promiscuity and overlapping specificities. On top of that, many of these enzymes such as zDHHC5 [40], require palmitoylation by other zDHHCs in order to function properly in a native environment. The complexity of this environment makes it difficult to dissect the biochemical mechanisms of zDHHC enzymes in the context of a cell. For example, siRNA knockdown of one zDHHC may indirectly reduce palmitoylation of a target protein because it acts as an upstream zDHHC which regulates membrane trafficking of the true enzyme of interest. False positive results may also occur in overexpression screens. zDHHC20 overexpression increases the co-immunoprecipitation of zDHHC5 with its substrate protein, phospholemman [40]. Overlapping substrate specificities may also create false negative results in knockdown screens if a second endogenous zDHHC, capable of palmitoylating the same substrate, is sufficient to compensate for the loss of the first. Thus, while *in vivo* and *in cellulo* methods can give important indications, they really cannot be used as confirmatory evidence for a zDHHC-substrate relationship. For these reasons, the development of *in vitro* methods for identifying true enzyme/substrate pairs is crucial for the validation of *in vivo* results and represents a key first step toward understanding protein S-acylation on a biochemical level.

One of the major barriers to *in vitro* reconstitution is the production of purified or semi-purified substrate and enzyme proteins. zDHHC enzymes, being eukaryotic integral membrane proteins, have the well-known challenges associated with eukaryotic transmembrane protein overexpression and purification. However, it is worthwhile to note that a large number of substrates are themselves transmembrane proteins, bringing in not only their own challenges of purification but also the subsequent challenge of reconstitution of substrate S-acylation with two different transmembrane proteins. Another challenge associated with substrate preparation is that the majority of them are complex eukaryotic proteins, often necessitating the use of eukaryotic hosts for expression and purification. However, this also implies that they would be S-acylated in the expression host and would have to be subsequently deacylated before they can be used in an *in vitro* substrate S-acylation assay.

Early studies by Linder and co-workers [29] used *in vitro* reconstitution to identify the mammalian homologues of Erf2 and Erf4 and validate their role in palmitoylation of H- and N-Ras. It is useful to point out here that Erf2 is the zDHHC enzyme and Erf4 is a regulatory cytosolic partner protein; the functional enzyme is the Erf2-Erf4 hetrodimeric complex. Sequence analysis implicated zDHHC9 and the regulatory subunit GCP16 as homologues of Erf2 and Erf4 respectively. These proteins were co expressed in sf9 cells and extracted with dodecyl-maltoside before subsequent purification by a series of affinity chromatography steps. Substrate proteins H- and N-Ras were also purified from sf9 cells. The authors noted that although H-Ras and N-Ras were S-acylated in sf9 cells, during the course of purification, these were probably hydrolysed owing to the action of thioesterases or action of reducing agents. Substrate proteins were incubated with zDHHC9/GCP16 in the presence of [^3^H]palmitoyl-CoA (figure 4*b*). The reaction was then quenched by addition of SDS loading buffer and run on a gel where the levels of palmitoylation were visualized by fluorography and subsequently quantified by scintillation spectroscopy on protein from excised gel bands.

As part of the first structural studies of full-length zDHHC enzymes, our laboratory also successfully demonstrated *in vitro* reconstitution of S-acylation by zDHHC20 and zDHHC15, with their respective substrates, GobX and SNAP25b [10]. zDHHC20 and zDHHC15 were purified from *Pichia pastoris*, and substrate proteins were purified from *E. coli*. GobX is an effector protein encoded by the intracellular bacterial pathogen, *Legionella pneumophila* [22]. Thus the choice of bacterial expression was a natural one. For SNAP25b, our laboratory had already worked out a method for its overexpression and purification from *E. coli* [41]. Since bacteria do not have the protein S-acylation enzymes, the substrate proteins in this case were not S-acylated during production. In this study, we used a fluorescently labelled palmitoyl-CoA analogue (NBD-palmitoyl-CoA) for in gel visualization of the acylated substrate.

*In vitro* reconstitution has also been used to obtain kinetic parameters of protein S-acylation for zDHHC2 and zDHHC3. An HPLC based assay was used to monitor the acylation of short, synthetic peptide consisting of residues Gly-Cys-Gly with an N-terminal myristoylation. The myristoyl group increases the hydrophobicity of the otherwise short peptide, presumably inducing partitioning into detergent micelles, thus bringing the peptide in close proximity to the zDHHC and allowing for the catalysis of acyl transfer. Acylation of the peptide was monitored and quantified by the shift in retention time over a reversed phase C4 column. One benefit of using this method is the ability to use native acyl-CoA, as opposed to fluorescent or radiolabelled analogues, allowing for more convenient use of acyl-CoAs with a variety of chain lengths and degrees of saturation. The assays revealed Vmax of 9.9 pmol^−1^ min^−1^ pmol and a Km of 1.3 µm myrGCG for zDHHC3 and Vmax of 5.2 pmol^−1^ min^−1^ pmol and a Km of 0.8 µm myrGCG for zDHHC2, indicating very similar rates of reaction and affinity for protein substrate between the two enzymes [42].

Our laboratory recently developed a hybrid of multiple previously validated techniques to demonstrate the first *in vitro* reconstitution of S-acylation of the SARS-CoV-2 Spike protein [35]. We used a click chemistry-based assay to demonstrate S-acylation of the C-terminal tail of the SARS-CoV-2 Spike protein. To avoid the complications with expressing, purifying and depalmitoylating full-length Spike protein from mammalian cell culture, we designed a bacterial expression construct that consisted of the proximal, intracellular C-terminal segment of Spike encompassing all 10 putative S-acylation sites, N-terminally modified with a myristoylation site and fused at its C-terminus to GFP. *In vitro* S-acylation assay was carried out with zDHHC enzymes purified from mammalian cells, and a terminal alkyne analogue of fatty acyl-CoA. Subsequently, the S-acylation of the substrate was detected by click chemistry-based attachment of a fluorophore at the terminal alkyne. We produced 7 different versions: one in which all Cys residues were mutated to Ala, and 6 constructs each with one cluster of cysteines introduced onto this Cysless background. We observed robust S-acylation activity with three different zDHHC enzymes and there was clear preference among the different cysteine clusters for S-acylation. Finally, although these cysteines are placed in close proximity to each other, there is notable selectivity among the different zDHHC members in their activities on these different sites. Remarkably, there was striking agreement between the *in cellulo* experiments and the results from the *in vitro* assays, which implied that our assay recapitulated the essential aspects of the Spike-zDHHC interactions inside the cell. Although S-acylation was first discovered in viral proteins [2] and has subsequently been shown to be important for many viruses including SARS-CoV-2 [8,43], this was the first successful reconstitution of *in vitro* S-acylation of a viral protein. The aforementioned experiments also provided a framework for designing *in vitro* assays for substrate S-acylation that could be used for high throughput screening and discovery of specific inhibitors of S-acylation of therapeutically suitable viral protein targets.

## 6. *In vitro* reconstitution and acyl-CoA selectivity

The zDHHC family of PATs are capable of accepting and transferring fatty acids of variable chain lengths and degrees of saturation to their respective protein substrates. Thanks to newly devised methods of reconstituting and analysing protein acylation, as well as enlightening new structural data, the mechanisms which drive the selectivity of acyl-CoAs among the various acyltransferases in general, and zDHHC enzymes specifically, have become much more clear in recent years. Though they are not representative of the entire S-acylated cellular proteome, one study conducted on platelet proteins found that, while the majority of thioester-linked fatty acids consisted of palmitate (about 74%), other fatty acids, such as stearate (C18:0) and oleate (C18:1) were also present [44]. Additionally, other studies have demonstrated that the diversity of acyl chain lengths observed on S-acylated proteins includes myristic acid (C14:0), palmitoleic acid (C16:1), linoleic acid (C18:2), arachidonic acid (C20:4) and eicosapentaenoic acid (C20:5) [45]; L [46]; P. [47]. As one might expect with such a diverse array of fatty acid substrates, it has been clearly demonstrated that different members of the zDHHC family have varying propensities for the assortment of acyl-CoA at their disposal [10,42,48]. In this section, we will discuss and analyse the variety of methods used to investigate the selectivity of acyl-CoAs amongst the zDHHC family of PATs.

In order to obtain a holistic understanding of the underlying structural and biochemical determinants of acyl chain selectivity, a variety of systems, methods, and detection techniques must be utilized. One of the first studies [42] to examine this aspect of zDHHC mechanism used partially purified forms of zDHHC2 and zDHHC3 in an assay which examined their propensity to become autoacylated by acyl-CoA with varying chain lengths and degrees/positions of saturation as well as their ability to transfer those same fatty acids onto substrate proteins. This was done by preloading the zDHHC with radiolabelled [^3^H]palmitoyl-CoA followed by the addition of non radiolabelled acyl-CoAs in the presence and absence of substrate protein and measuring by scintillation spectroscopy, the ability of the nonlabelled acyl-CoAs to compete. The results from this study demonstrated two key findings. First, by showing that the level of autoacylation for both enzymes corresponded to the level of substrate acylation across all reaction conditions, which demonstrated that autoacylation is a reaction intermediate, the results lent strong credence to the ping-pong mechanism hypothesis (depicted in figure 2). Second, the results indicated stark differences is the abilities of individual zDHHCs to use acyl-CoAs of varying chain lengths. Specifically, they showed that while zDHHC2 could use fatty acid chains with up to at least 20 carbons, zDHHC3 demonstrated a narrower range of length tolerance, incorporating and transferring only 14- and 16-carbon fatty acids [42].

With general interest in zDHHC enzymes intensifying due to their increasing relevance to multiple human diseases such as cancer [49,50] and Huntington's disease [51], researchers began seeking out more accessible methods for detecting autoacylation without the need for radiolabelled compounds. The fluorescence-based, coupled-enzyme assay, developed by Hamel *et al*. [52], allowed for indirect detection of autoacylation by quantification of the production of reduced CoA (CoASH). This is accomplished by coupling the initial reaction catalysed by the zDHHC enzymes with a secondary reaction in which fluorescent NADH is produced from NAD^+^ during the conversion of α-ketoglutarate to succinyl-CoA by α-ketoglutarate dehydrogenase. The major advantage of this methods is in its applicability to high throughput, microplate formats, which allows for the quick, accurate and simultaneous assessments of enzymatic activity using a variety of acyl-CoA substrates in a range of conditions in. This method was used to determine kinetic parameters for the Erf2-Erf4 complex (the yeast orthologue of the human zDHHC9-GCP16 complex) and was also able to show that it demonstrates a preference for myristoyl-CoA and palmitoleoyl-CoA [52]. In addition to in depth kinetic parameter, this method can also be used to quickly assess protein functionality as a benchmark for initiating structural studies [10].

## 7. Conclusion

Since the discovery of protein S-acylation, our knowledge of the cellular machinery that drives this modification and the repertoire of proteins that are targets of S-acylation have undergone an explosive growth. Discoveries about how protein S-acylation intersects with protein localization, function and how that, in turn, affects cellular physiology continue to add to the rich expanse of literature on the subject. Yet, discrete mechanistic understanding of interactions between zDHHC enzymes and their substrates has been sorely lacking. We posit that at the heart of this lack of knowledge is the paucity of experiments reconstituting the S-acylation of substrate proteins with purified components in a test tube. The 23 zDHHC enzymes and over 4000 substrates in humans alone potentially facilitate *thousands* of zDHHC-substrate interactions, of which we currently have little to no understanding from this perspective [53]. This situation presents an obvious challenge, owing to the inherent difficulties in reconstitution of enzymatic reactions with integral membrane enzymes and peripheral or integral membrane substrates. Notable advancements have been made recently, but future investigations about this aspect of protein S-acylation will probably be critical to our understanding of protein S-acylation, from the atomic to cellular level.

## Data Availability

This article has no additional data.
